# Survey data of COVID-19-related Knowledge, Risk Perceptions and Precautionary Behavior among Nigerians

**DOI:** 10.1016/j.dib.2020.105685

**Published:** 2020-05-08

**Authors:** Peter O. Olapegba, Steven K. Iorfa, Samson O. Kolawole, Rotimi Oguntayo, Joshua C. Gandi, Iboro F.A. Ottu, Olusola Ayandele

**Affiliations:** a Department of Psychology, University of Ibadan, Ibadan, Nigeria; b Department of Psychology, University of Nigeria, Nsukka, Nigeria; c Department of Psychology, Nigeria Police Academy, Wudil, Kano, Nigeria; d Department of Psychology, University of Ilorin, Ilorin, Nigeria; e Department of Psychology, University of Jos, Jos, Nigeria; f Department of Psychology, University of Uyo, Uyo, Nigeria; g Department of General Studies, The Polytechnic, Ibadan, Nigeria

**Keywords:** Survey data, COVID-19, Knowledge, Risk Perceptions, Precautionary Behavior, Nigeria

## Abstract

In response to the global call for strategic information to understand the novel coronavirus, the dataset presented in this paper is an examination of COVID-19-related knowledge, risk perceptions and precautionary health behavior among Nigerians. The data were generated during the COVID-19 lockdown in the country through a survey distributed via an online questionnaire, assessing socio-demographic information (7 items), knowledge (5 items), information sources (1 item), risk perception (9 items), expected end of lockdown (1 item), and COVID-19 precautionary health behavior (10 items), from 28th March to 4th April, 2020, gathering a total of 1,357 responses. A combination of purposive and snowball techniques helped to select the respondents via Whatsapp and Facebook from 180 cities/towns in the 6 geopolitical zones of Nigeria. The survey data were analyzed using descriptive statistics. The entire dataset is stored in a Microsoft Excel Worksheet (xls) and the questionnaire is attached as a supplementary file. The data will assist in curbing the Coronavirus pandemic by offering evidence for strategic and targeted interventions as well as health policy formulations and implementation.

Specifications table

**Table:** 

Subject	Public Health
Specific subject area	Health Psychology, Social psychology
Type of data	Primary data, Tables
How data were acquired	Data was obtained using questionnaires hosted on an online survey survey platform (google forms). The questionnaire is included in this article and may be accessed online via the following link https://forms.gle/RjrcYpAkVQbhnN2s6
Data format	Raw, Analyzed, Filtered (descriptive statistics)
Parameters for data collection	The data for the survey were obtained from respondents in 180 Nigerian cities/towns with internet access.
Description of data collection	A combination of purposive and snowball techniques helped to select the respondents via Whatsapp and Facebook from all the 6 geopolitical zones of Nigeria.
Data source location	Surveys were conducted in all six (6) geopolitical zones of Nigeria
Data accessibility	Dataset is uploaded on Mendeley
	Repository Name: Mendeley
	Direct URL to data: https://data.mendeley.com/datasets/k4nch8rrt5/draft?a=d5e854de-306d-48e7-a88e-71f2ccab80ff

## Value of the data

• The data represent the first and one of the largest pools so far for the exploration of COVID-19-related knowledge, information sources, risk perception, expected end of lockdown and precautionary health behavior among Nigerians.
• The data can be compared with similar studies on knowledge, information sources, risk perception, expected end of lockdown and COVID-19 precautionary health behavior from other countries around the world and may serve as a heuristic basis for further insight into the phenomena of the COVID 19.
• The data can be statistically analyzed to examine the relationships between socio-demographics, knowledge, information sources, risk perception, expected end of lockdown, and COVID-19 precautionary health behaviors.
• The details of the analyzed data are beneficial for preventing and curbing the spread of COVID-19 and the data can assist with planning for public health interventions as well as policy formulation and implementation.

## 1 Data description

This data set provides information on COVID-19-related knowledge, information sources, risk perception, expected end of lockdown and precautionary health behavior among Nigerians. The obtained raw data used for each table is stored in a Microsoft Excel Worksheet (xls). Items 1-7 elicit respondents’ gender, age, marital status, ethnicity, educational qualification, religion and perceived financial situation. Items 8-12 assessed respondents’ COVID-19 knowledge and item 13 their sources of new information about COVID-19. Items 14 – 23 and 24 – 32 measured their of COVID-19 perceived threat and preventive behavior respectively while item 33 revealed “how soon they expected things to return to normal.” Demographic characteristics of respondents are presented in Table 1. The detailed assessments of responses on COVID-19-related knowledge, information sources, risk perception, expected end of lockdown and precautionary health behavior by residents of Nigeria are depicted in Table 2, Table 3, Table 4, Table 5, Table 6. The data presented below revealed that most Nigerians had sufficient knowledge on COVID-19 (Mean 4.15; S.D. = 0.77), perceived it as a threat and engaged in Precautionary Behavior.

**Table 1:** Descriptive statistics of sample characteristics (n = 1,357).

Variables	Categories	Frequency	Percent	Statistics
Age	15 – 24 years	736	54.24	Mean 26.85, S.D. = 9.17
	25 – 34 years	401	29.55	
	35 and above	220	16.21	
Gender	Female	570	42.00	
	Male	787	58.00	
Educational qualification	High School	349	25.72	
	Diploma	313	23.07	
	Degree	421	31.02	
	Higher degree	242	17.83	
	Others	32	2.36	
Perceived Socio-Economic Class	Lower	246	18.13	
	Lower Middle	479	35.30	
	Upper Middle	387	28.52	
	Upper	245	18.05	
Relationship Status	Single/Not dating	560	41.27	
	Single/but dating	524	38.61	
	Married	269	19.82	
	Separated/divorced/widowed	4	0.29	
Ethnic Grouping	Hausa-Fulani	138	10.17	
	Igbo	115	8.47	
	Yoruba	929	68.46	
	Other ethnicities	175	12.90	
Religion	Christianity	842	62.05	
	Islam	506	37.29	
	Others	9	0.66

**Table 2:** Descriptive statistics on COVID-19-related knowledge (Mean is 4.15, S.D. = 0.77).

Number of correct answers	Frequency	Percent
None	1	0.07
One	4	0.29
Two	33	2.43
Three	181	13.34
Four	667	49.15
Five	471	34.71
Total	1357	100

**Table 3:** Descriptive statistics of COVID-19-related information sources (n = 1,357).

Information sources	Frequency	Percent
Mass media	1106	81.5
Social media	953	70.23
The Internet	835	61.53
Health workers	614	45.25
Family & friends	507	37.36
Government	371	27.34

**Table 4:** Descriptive statistics on the practice of COVID-19-related precautionary health behaviors.

Items	Responses			Statistics	
Since the start of this Coronavirus pandemic, (1=strongly disagree, 7=strongly agree)	Disagreement (1-3)	undecided (4)	Agreement (5-7)	Mean	S.D.
It really bothers me when people sneeze without covering their mouths	8.99	8.40	82.61	6.02	1.60
I prefer to use hand sanitizer or wash my hands after shaking someone's hand	10.98	7.96	81.06	5.91	1.74
I avoid touching door handles and stair case railing at public locations	15.11	9.14	75.76	5.64	1.87
I dislike wearing face mask	52.98	13.71	33.31	3.42	2.33
I want people's temperature to be taken before they enter public places	21.00	11.27	67.72	5.26	2.12
I don't mind going to very crowded places	82.54	6.12	11.35	2.03	1.77
I would self-isolate myself at home if needed	10.10	5.16	84.75	6.05	1.70
I frequently use hand sanitizer	18.20	10.61	71.19	5.47	2.01
I avoid going to public places	12.16	8.84	79.00	5.80	1.74
I have changed the way I live my life because of Coronavirus	15.92	7.22	76.86	5.62	1.96

**Table 5:** Descriptive statistics on COVID-19-related risk perception (n = 1,357).

Items	Responses			Statistics	
(1=Not at all, 7=Extremely)	Not at all (1-3)	undecided (4)	Extremely (5-7)	Mean	S.D.
Compared to most people of my age, my risk of getting Coronavirus is	54.75	19.23	26.01	3.29	2.09
What level of threat do you think the Coronavirus pandemic poses to your studies?	10.17	6.56	83.27	5.93	1.66
The likelihood of my getting Coronavirus is	69.12	13.71	17.17	2.69	1.97
How likely do you think people in Nigeria (or your country) are to contract the Coronavirus?	17.46	15.62	66.91	5.15	1.83
How likely do you think your colleagues are to contract the Coronavirus?	46.43	21.74	31.83	3.66	1.98
How likely do you think people in your present location are to contract the Coronavirus?	54.83	16.06	29.11	3.37	2
How worried are you about contracting the Coronavirus?	31.83	11.79	56.37	4.72	2.26
How likely do you think you would meet someone who is infected with Coronavirus?	62.64	15.48	21.89	2.99	1.98
How worried are you that your family members or friend might be infected by Corona Virus?	42.74	13.04	44.22	4.09	2.31

**Table 6:** Descriptive statistics of respondents’ expected end of COVID-19 lockdown (n = 1,357).

Expected end	Frequency	Percent
April-May 2020	1004	73.99
June-August 2020	106	7.81
September-December 2020	110	8.10
I don't know when	137	10.10
Total	1357	100.00

## 2 Survey design, materials, and methods

The research adopted a descriptive survey design to evaluate the dataset on the knowledge, information sources, risk perception, expected end of lockdown and COVID-19 precautionary health behavior in Nigeria. This dataset included 1357 responses collected between 28^th^ March and 4^th^ April, 2020 from the 6 geopolitical zones of Nigeria. Ethical approval was obtained from the Faculty of Social Sciences Ethical Board, University of Ibadan. Respondents’ participation was completely consensual, anonymous and voluntary.

The researchers used an online questionnaire to collect data for this survey (See Appendix). Eight items (section A) elicit respondents’ demographic information, including their gender, age, marital status, ethnicity, educational qualification, religion and perceived financial situation. Five items (Section B) adapted from [1] and validated by [2] assessed respondents’ COVID-19 knowledge by summing correct responses across item 1, source of COVID-19, (correct = [d]), item 2, transmission of COVID-19, (correct = [a], [b] and [c]), item 3, prevention of COVID-19, (correct = [b], [c], [e] and [g]), item 4, symptoms of COVID-19, (correct = [a], [b] and [g]), and item 5, awareness of COVID-19 fatality, (correct = [a]), generating a maximum possible score of five (norm is set at 3 to indicate moderate level of COVID-19 knowledge[2] . One item (Section C) assessed sources of new information about COVID-19, and nine items (section D) adapted from [3] measured perceived threat of COVID-19 (Cronbach Alpha is 0.76), while ten items (Section E) adapted from [4,5] measured preventive behavior (Cronbach Alpha is 0.75) and One item (Section F) asked respondents “how soon they expected things to return to normal”

The respondents’ demographics, COVID-19-related knowledge, knowledge, information sources, risk perception, expected end of lockdown and precautionary health behavior were analyzed using frequencies, percentages, means, and standard deviations. Correlation analyses, to understand the relationships between demographics, knowledge, information sources, risk perception, expected end of lockdown and COVID-19 precautionary health behavior, were conducted using Statistical Package for Social Sciences Version 20.

## Appendix Supplementary materials

Image, application 1

Image, application 2
