# Supplementary material for: Survey data of COVID-19-related Knowledge, Risk Perceptions and Precautionary Behavior among Nigerians
Source: Data Brief. 2020 May 8;30:105685. doi: 10.1016/j.dib.2020.105685 (PMC7206440; doi:10.1016/j.dib.2020.105685)
Supplement: Supplementary file 2 [file mmc2.docx]

# Questionnaire

## **Section A: Socio-demographics**

Present location: _______________________

1. Gender: 1, Male; 2, Female

2. Age____ years

3. Relationship status: 1, Single; 2, Dating; 3, Married; 4, Others

4. Ethnicity:__________ 1, Hausa; 2, Igbo; 3, Yoruba; 4, Others

5. Highest Educational Qualification: 1, High school; 2, Diploma; 3, Degree; 4, Postgraduate; 5, Others

6. Religion: 1, Christianity; 2, Islam; 3 Traditional & Others

7. *Considering your own income and the income from any other people who help you*, how would you describe your overall personal financial situation? 1, don’t meet basic needs; 2, just meet basic needs; 3,meet needs with a little left; 4,live comfortably

## **Section B: Knowledge of Coronavirus**

8. To the best of your knowledge, the novel Coronavirus is: (*You may choose more than one).*

(a) a biological weapon designed by the government of China

(b) a virus designed by pharmaceutical industry to sell their drugs

(c) an exaggeration by news media to cause fear and panic

(d) a severe illness transmitted to people from wild animals

(e) a plague caused by sins and unbelief of human being

(f) designed to reduce or control the population

(g) a biological weapon designed by the USA government

9. The Coronavirus is typically spread (i.e., passed from person-to-person) by which means? (*You may choose more than one*).

(a) contact with airborne droplets via breathing, sneezing, or coughing,

(b) kissing, hugging, sex or other sexual contact

(c) eating of contaminated water or food

(d) touching contaminated objects or surfaces

10. Coronavirus can be prevented by

(a) the hot weather of Africa

(b) regular hand washing and social distancing

(c) taking chloroquinne capsules and antibiotics

(d) fumigation and spraying bus stops and other public places

(e) eating of traditional African food and soup

(f) closing schools and cancelling mass gathering events

(g) consuming gins, garlic, ginger and herbal mixtures

(h) Disinfecting contaminated surfaces

11. The most important symptoms of COVID19 / Coronavirus are: *(You may choose more than one).*

(a) cough

(b) fever

(c) fatigue

(d) sneezing

(e) sore throat

(f) muscle pain

(g) shortness of breath

(h) I do not know any symptoms of COVID19/Coronavirus

12. Do you think it is possible to die from the Coronavirus?

(a) yes,

(b) no,

(c) I do not know

13. Which of the following sources have you received new information about the Coronavirus? *(You may choose more than one)*.

(a) The mass media (television, newspapers, radio etc.)

(b) the internet (Google, Wikipedia, etc.)

(c) Health workers (doctors, nurses, pharmacist, NCDC, etc.)

(d) Government officials (governors, ministers, commissioners, etc.)

(e) Friends and family members

(f) Social media (Whatsapp, Facebook, Instagram, Twitter etc.)

(g) other: …

## **Section C: Precautionary Behavioral**

*Since the start of this Coronavirus pandemic, (1 = strongly disagree, 7 = strongly agree)*

14. It really bothers me when people sneeze without covering their mouths

15. I prefer to use hand sanitizer or wash my hands after shaking someone’s hand

16. I avoid touching door handles and stair case railing at public locations

17. I dislike wearing face mask because it looks somehow

18. I want people’s temperature to be taken before they enter public places

19. I don’t mind going to very crowded places

20. I would self-isolate myself at home if needed

21. I frequently use hand sanitizer after shaking someone’s hand

22. I avoid going to public places

23. I have changed the way I live my life because of Coronavirus

## Section D: Risk Perceptions of COVID-19

24. Compared to most people of my age, my risk of getting Coronavirus is *(1 = extremely low, 7 = extremely high).*

25. What level of threat do you think the Coronavirus pandemic poses to your studies? *(1 = no threat at all, 7 = extremely high level of threat)*

26. The likelihood of my getting Coronavirus is *(1 = not at all likely, 7 = extremely likely)*

27. How likely do you think people in Lagos are to contract the Coronavirus? *(1 = not at all likely, 7 = extremely likely)*

`28. How likely do you think people in Ibadan are to contract the Coronavirus? *(1 = not at all likely, 7 = extremely likely)*

29. How likely do you think people in your hometown are to contract the Coronavirus? *(1 = not at all likely, 7 = extremely likely)*

30. How worried are you about contracting the Coronavirus? *(1 = not at all worried, 7 = extremely worried)*

31. How likely do you think you would meet someone who is infected with Coronavirus *(1 = not at all likely, 7 = extremely likely)*

32. How worried are you that your family members or friend might be infected by Corona Virus (*1 = not at all worried, 7 = extremely worried)*

33. How soon do you expect the Coronavirus holiday to end /things to return to normal?

(a) April-May 2020

(b) June-August 2020

(c) September-December 2020

(d) January 2021

(e) I don’t know
